# Codelivery of BCL2 and MCL1 Inhibitors Enabled by Phenylboronic Acid‐Functionalized Polypeptide Nanovehicles for Synergetic and Potent Therapy of Acute Myeloid Leukemia

**DOI:** 10.1002/advs.202204866

**Published:** 2023-01-22

**Authors:** Jiguo Xie, Xiaofei Zhao, Peng Zhang, Yueyue Zhang, Ru Cheng, Zhiyuan Zhong, Chao Deng

**Affiliations:** ^1^ Biomedical Polymers Laboratory and Jiangsu Key Laboratory of Advanced Functional Polymer Design and Application College of Chemistry Chemical Engineering and Materials Science and State Key Laboratory of Radiation Medicine and Protection Soochow University Suzhou 215123 P. R. China

**Keywords:** acute myeloid leukemia, BCL2 inhibitor, B—N coordination, boronic ester bond, MCL1 inhibitor, synergetic therapy

## Abstract

Acute myeloid leukemia (AML) is the most refractory hematologic malignancy characterized by acute onset, rapid progression, and high recurrence rate. Here, codelivery of BCL2 (ABT199) and MCL1 (TW37) inhibitors using phenylboronic acid‐functionalized polypeptide nanovehicles to achieve synergetic and potent treatment of AML is adopted. Leveraging the dynamic boronic ester bonds, B—N coordination, and *π*–*π* stacking, the nanovehicles reveal remarkably efficient and robust drug coencapsulation. ABT199 can induce a series of pro‐apoptotic reactions by promoting the dissociation of the pro‐apoptotic protein Bim from BCL2, while the released Bim is often captured by MCL1 protein overexpressed in AML. TW37 has a strong inhibitory ability to MCL1, thereby can restrain the depletion of Bim protein. Dual inhibitor‐loaded nanoparticles (NPAT) reveal excellent stability, acid/enzyme/H_2_O_2_‐triggered drug release, and significant cytotoxicity toward MOLM‐13‐Luc and MV‐411 AML cells with low half maximal inhibitory concentrations of 1.15 and 7.45 ng mL^−1^, respectively. In mice bearing MOLM‐13‐Luc or MV‐411 AML cancer, NPAT reveal significant inhibition of tumor cell infiltration in bone marrow and main organs, potent suppression of tumor growth, and remarkably elevated mouse survival. With facile construction, varying drug combination, superior safety, synergetic efficacy, the phenylboronic acid‐functionalized smart nanodrugs hold remarkable potential for AML treatment.

## Graphical abstract

Phenylboronic acid‐functionalized polypeptide nanonvehicles achieve nearly quantitative coencapsulation and acid/enzyme/H_2_O_2_‐triggered release of both BCL2 (ABT199) and MCL1 (TW37) inhibitors. In MOLM‐13‐Luc and MV‐411 acute myeloid leukemia models, dual inhibitor‐loaded nanodrugs reveal significant inhibition of tumor cell infiltration in bone marrow, lung, liver, spleen, and peripheral blood, potent suppression of tumor growth, and remarkably elevated mouse survival.

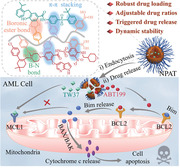

## 1 Introduction

Acute myeloid leukemia (AML) is an aggressive hematologic malignancy with a high level of immature malignant cells and impaired normal hematopoiesis derived from clonal proliferation of myeloid precursors.^[^ ^1^ ^]^ With the characteristics of acute onset, rapid progression, and high recurrence rate, AML has emerged as the most refractory among various leukemias.^[^ ^2^ ^]^ “7+3” therapy including continuous infusion of cytarabine for 7 d and i.v. administration of daunorubicin on the initial 3 d has been recognized as the treatment paradigm of AML.^[^ ^3^ ^]^ Although the therapy affords complete remission (CR) for 60%–85% of AML patients with an age of lower than 60, the five‐year relative survival for older patients (≥65 years of age) is around 7%.^[^ ^4^ ^]^ Of note, AML is a disease of the elderly, in which one‐third of patients are older than 75 years and the median age at diagnosis is 67 years old.^[^ ^5^ ^]^ Recently, a liposomal formulation of cytarabine and daunorubicin at a molar ratio of 5:1 (CPX‐351) was developed and demonstrated obviously promoted median overall survival (OS, 9.56 vs 5.95 months) and overall remission rate (47.7% vs 33.3%) versus “7+3” in older patients (60–75 years of age) with secondary AML.^[^ ^6^ ^]^ Although chemotherapeutics revealed certain therapeutic outcomes, massively adverse toxicity and limited efficacy hamper their clinical applications.^[^ ^7^ ^]^

Comparing with chemotherapeutics, small molecule inhibitors with high specificity, low off‐target toxicity, and significant anticancer effects have been explored for AML treatment.^[^ ^8^ ^]^ Several inhibitors including midostaurin, gilterinib, and ABT199 have been recently approved by the U.S. Food and Drug Administration (FDA) for AML treatment.^[^ ^9^ ^]^ In a phase IIB trial, midostaurin with activity against the FMS‐like tyrosine kinase 3 receptor (FLT3) demonstrated well tolerance and one partial response (PR) in thirty‐five FLT3‐mutated patients receiving 100 mg twice daily.^[^ ^10^ ^]^ Anti‐apoptotic B‐cell lymphoma 2 (BCL2) protein commonly overexpressed in leukemia cells is associated with poor prognosis and treatment resistance in AML patients.^[^ ^11^ ^]^ ABT199, a highly selective BCL2 inhibitor afforded an overall response rate of 19% and median OS of 4.7 months in patients with relapsed/refractory AML.^[^ ^12^ ^]^ The moderate therapeutic outcomes highlight the compulsion of combination therapy of small molecule inhibitors with other agents like chemical agents. In a phase III study, the combination of midostaurin with standard chemotherapy in patients with FLT3‐mutat AML afforded significant survival benefit with a remarkable elevated median OS (74.7 vs 25.6 months), and reduced cumulative incidence of relapse.^[^ ^13^ ^]^ Similarly, the addition of ABT199 to decitabine or azacitidine exhibited favorable overall response rate with a CR rate of around 67% in treatment‐naive, elderly patients with AML.^[^ ^14^ ^]^ Recently, different small molecule inhibitors with diverse potency and specificity were combined to treat heterogeneous AML. For example, the combination of MCL1, FLT3, and CDK inhibitors with BCL2 inhibitor generated synergistic potency and improved antileukemic activity against cell lines and patient samples in preclinical experiments.^[^ ^15^ ^]^ However, the inhibitor combination is tortured by their intrinsically poor bioavailability and transient target regulation, which typically necessitates a continuous and repeated treatment schedule.

**Scheme 1:**
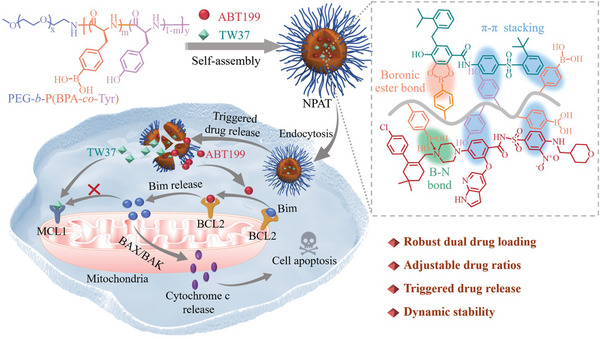
Schematic illustration of codelivery of BCL2 (ABT199) and MCL1 (TW37) inhibitors by nanovehicles formed from poly(ethylene glycol)‐*b*‐poly(l‐boronophenylalanine‐*co*‐l‐tyrosine) (PEG‐*b*‐P(BPA‐*co*‐Tyr)) for synergetic and potent therapy of acute myeloid leukemia (AML).

In this contribution, we have designed and developed nanovehicles from poly(ethylene glycol)‐*b*‐poly(l‐boronophenylalanine‐*co*‐l‐tyrosine) (PEG‐*b*‐P(BPA‐*co*‐Tyr)) for efficient coencapsulation and responsive release of both BCL2 (ABT199) and MCL1 (TW37) inhibitors, achieving synergetic and potent treatment of AML (**Scheme** **1**). BPA and Tyr segments in the nanovehicles would bind different drugs via boronic ester bonds, B—N coordination, and *π*–*π* stacking, which have been comprehensively utilized for robust drug loading and nanovehicle stabilization.^[^ ^16^ ^]^ Besides, polypeptides provide decent biocompatibility and enzymatic degradation that facilitates responsive drug release.^[^ ^17^ ^]^ ABT199 can induce a series of pro‐apoptotic reactions by promoting the dissociation of the pro‐apoptotic protein Bim from BCL2,^[^ ^11^ ^]^ and TW37 can downregulate MCL1^[^ ^18^ ^]^ that is often highly expressed in AML and thus hamper the binding of released Bim with MCL1. The released Bim can cause homo‐oligomerization and activation of BAX and BAK, and thus increase the permeabilization of mitochondrial outer membrane, leading to the release of apoptogenic factors like cytochrome c from mitochondria into cytoplasm. The released cytochrome c can activate caspase‐9/‐3 pathway, and induce cell apoptosis. In mice bearing MOLM‐13‐Luc and MV‐411 AML cancers, dual drug‐loaded nanoparticles (NPAT) revealed significant inhibition of cancer cell infiltration in bone marrow (BM) and main organs, potent tumor suppression, and remarkably prolonged survival.

## 2 Results and Discussion

### 2.1 BCL2 and MCL1 Overexpression in AML, Cytotoxicity of BCL2 and MCL1 Inhibitors

**Figure 1:**
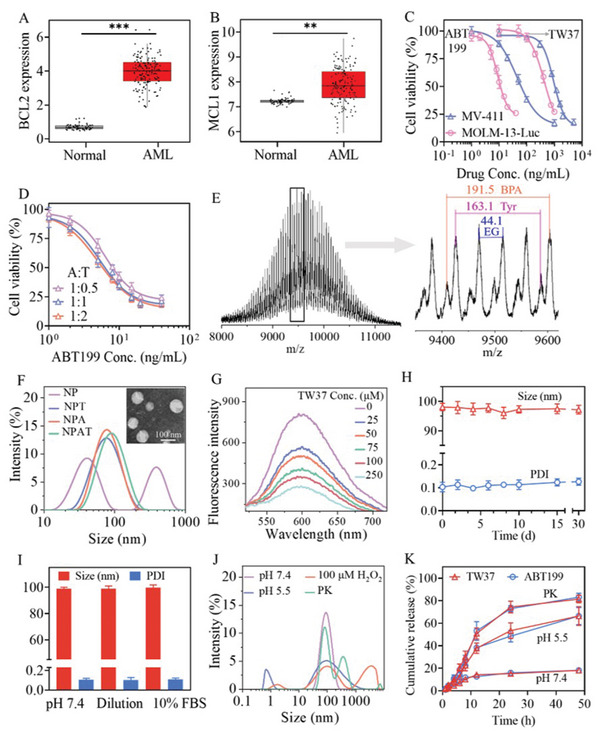
The expression of A) BCL2 and B) MCL1 genes in AML patients (*n* = 132) and healthy people (*n* = 70) isolated from The Cancer Genome Atlas (TCGA) and Genotype‐Tissue Expression (GTEx), respectively. The statistical significances were assessed by the one‐way ANOVA, **p < 0.01, ***p < 0.001. C) Cell viability of MOLM‐13‐Luc and MV‐411 cell lines treated with free ABT199 or TW37 for 48 h (*n* = 6). D) Cell viability of MOLM‐13‐Luc treated with dual drugs at different ABT199/TW37 weight ratios (1:0.5, 1:1, 1:2) for 48 h (*n* = 6). (E) MALDI‐TOF spectrum of PEG‐*b*‐P(BPA‐*co*‐Tyr) (Table 1, entry 1). F) Size distribution of NP, NPT (Table S2, Supporting Information, entry 1), NPA (Table S2, Supporting Information, entry 5), and NPAT (Table 2, entry 1); inset: TEM image of NPAT. G) Competitive binding analysis of alizarin red S (ARS). The fluorescence intensities of the ARS/PEG‐*b*‐P(BPA‐*co*‐Tyr) complex at an ARS/copolypeptide molar ratio of 1:1 was reduced by competitive binding with TW37. H) Size and PDI changes of NPAT at 1.0 mg mL^−1^ against long‐term storage (*n* = 3). I) Stability of NPAT at 1.0 mg mL^−1^ against 10% FBS and 100‐fold dilution (*n* = 3). J) Bioresponsivity of NPAT in PBS (pH 5.5), PBS with 100 × 10^−6^ m H_2_O_2_ or proteinase K (PK, 12 units mL^−1^). K) In vitro drug release profiles from NPAT under acidic condition (pH 5.5) or in the presence of PK (12 units mL^−1^) (*n* = 3).

A bioinformatics analysis based on The Cancer Genome Atlas (TCGA) and Genotype‐Tissue Expression (GTEx) databases revealed that the expression level of anti‐apoptotic BCL2 and MCL1 genes in bone marrow of AML patients (*n* = 132) was significantly higher than that in normal bone marrow (*n* = 70) (**Figure** **1**A,B), in which the BCL2 expression in AML patients was around 10.7‐fold higher than that in healthy people. Thus, BCL2 and MCL1 are closely associated with the onset and progression of AML, and the inhibition of their expression or activity might provide an exclusive strategy for AML treatment.

In MOLM‐13‐Luc AML cell line, both BCL2 (ABT199) and MCL1 (TW37) inhibitors revealed remarkable cytotoxicity with half‐maximal inhibitory concentrations (IC_50_) of 8.89 and 430.6 ng mL^−1^, respectively (Figure 1C). Combining ABT199 and TW37 significantly increased cytotoxicity toward MOLM‐13‐Luc cells (Figure 1D) and presented an IC_50_ of 5.08 ng mL^−1^ when the ABT199/TW37 (A:T) weight ratio was 1:1 (Table S1, Supporting Information). Of note, the cytotoxicity could be facilely controlled by changing the drug weight ratios, and all dual‐drug formulations revealed remarkably synergistic cytotoxicity with combination indices (CI) of lower than 0.72. Similarly, ABT199, TW37, and their combinations afforded obvious cytotoxicity in MV‐411 AML cells, and the dual drugs at weight ratios (A:T) ranging from 1:0.5 to 1:2 afforded obviously synergistic antitumor activity (Figure S1 and Table S2, Supporting Information), further corroborating the inhibition of both BCL2 and MCL1 might be beneficial for AML treatment.

### 2.2 Construction and Characterization of Dual Inhibitor‐Loaded Nanoparticles (NPAT)

NP was constructed through the self‐assembly of PEG‐*b*‐P(BPA‐*co*‐Tyr) copolymer that was synthesized by simultaneous copolymerization of BPA‐NCA and Tyr‐NCA monomers in the presence of PEG‐NH_2_ (*M*_n_ = 5.0 kg mol^−1^) as previous report (Scheme S1, Supporting Information).^[^ ^19^ ^] 1^H NMR spectrum of PEG‐*b*‐P(BPA‐*co*‐Tyr) discerned characteristic signals of PEG (*δ* 3.50), BPA (*δ* 7.22, 7.68), and Tyr (*δ* 6.61, 6.95) (Figure S2A, Supporting Information). The number of BPA and Tyr units could be acquired by comparing the integrals of signal at *δ* 3.50 (methylene of PEG) with those at *δ* 7.68 (benzene ring of BPA) and *δ* 6.61 (benzene ring of Tyr), respectively. Moreover, the content of Tyr and BPA moieties could be facilely controlled by adjusting the feed ratios of monomers to PEG‐NH_2_ initiator (**Table** **1**), corroborating that the NCA copolymerization proceeded in a controlled manner and copolypeptides with tailored chain length and functionalities could be readily obtained. MALDI‐TOF and gel permeation chromatography (GPC) measurements revealed that PEG‐*b*‐P(BPA‐*co*‐Tyr) had similar molecular weights to those measured by ^1^H NMR and narrow polydispersities with an *M*_w_/*M*_n_ of around 1.10 (Figure 1E, Table 1).

**Table 1:** Synthesis of PEG‐*b*‐P(BPA‐*co*‐Tyr) copolymers

Entry	*M*_n_ [kg mol^−1^]			*M*_w_/*M*_n_ ^c)^	Yield [%]
	Design	^1^H NMR^a)^	MALDI‐TOF^b)^		
1	5.0‐4.0‐1.0	5.0‐3.9‐1.0	9.6	1.10	83
2	5.0‐3.0‐2.0	5.0‐3.0‐1.8	10.1	1.07	78
3	5.0‐2.0‐3.0	5.0‐1.9‐2.7	9.5	1.18	80

^a)^ Calculated from ^1^H NMR;

^b)^ Measured by MALDI‐TOF (matrix: trans‐2‐(3‐(4‐tertbutylphenyl)‐2‐methyl‐2‐propenylidene) malononitrile (DCTB)/CF_3_COONa^+^ (v/v = 9/1));

^c)^ Determined by GPC (eluent: DMF, flow rate: 0.8 mL min^−1^, 40 °C; standard: poly(methyl methacrylate)).

In order to maximize the drug loading, PEG‐*b*‐P(BPA‐*co*‐Tyr) (*M*_n_ = 5.0‐3.9‐1.0 kg mol^−1^) with the highest BPA content was employed to fabricate nanovehicles. Dynamic light scattering (DLS) measurement revealed that blank nanoparticle had broad size distribution at 10–100 and 200–800 nm (Figure 1F), indicating NP without drugs was not stable. The inferior stability of NP is likely owing to the partial hydrophilicity of BPA and Tyr moieties located in the nanoparticles cores. Through a solvent‐exchange method, TW37 or ABT199 was facilely encapsulated into nanoparticles (NPT, NPA). Both NPA and NPT presented high drug loading efficiency (DLE) of over 92% and decent drug loading content (DLC) up to 48.0 wt% (Table S3 and Figure S3A–D, Supporting Information). Upon coloading of TW37 and ABT199, NPAT revealed nearly quantitative loading of ABT199 (DLE > 97.6%) when the theoretical DLC ranged from 10 to 25 wt% (**Table** **2**, Figure S3E, Supporting Information). The remarkably high drug loading capacity could be attributed to the formation of dynamic boronic ester bonds, *π*–*π* stacking, B—N coordination, and hydrophobic interactions between drugs (TW37, ABT199) and amino acid moieties (BPA, Tyr), which has been employed to achieve decent drug encapsulation in varying delivery systems.^[^ ^20^ ^]^ Notably, the strong fluorescence at 600 nm (Figure S4, Supporting Information) derived from the complex of alizarin red S (ARS) with PEG‐*b*‐P(BPA‐*co*‐Tyr) via boronic ester bonds could be significantly decreased by TW37, and increasing TW37 concentrations generated weaker fluorescence (Figure 1G), verifying that TW37 competed over ARS and formed stronger boronic ester bonds with BPA moieties of copolypeptides. In addition, ^11^B NMR revealed around 8 ppm signal shift of ^11^B in PEG‐*b*‐P(BPA‐*co*‐Tyr) after mixing with ABT199 (Figure S5A, Supporting Information), indicating that the existence of B‐N coordination between ABT199 and polypeptide segments. Besides, UV spectra revealed an obvious redshift from 425 to 435 nm for ABT199 upon encapsulating into NP (Figure S5B, Supporting Information). Meanwhile, 2D ^1^H‐^1^H NOESY NMR revealed clear cross peaks at *δ* 6.5–7.8 (Figure S5C, Supporting Information) for NPA, which combining with significant red shift corroborates the existence of strong intermolecular *π*–*π* stacking between drugs and polypeptides. In addition, boron‐free nanoparticles (NP‐BF) formed from PEG‐*b*‐PTyr copolymer was employed to explore the effect of supramolecular interactions on drug loading, and the results revealed that NP‐BF exhibited significantly lower loading efficiency for both TW37 (15.6% vs 92.6%) and ABT199 (85.7% vs 98.2%) at theoretical drug loading contents of 10 wt% for TW37 and 25 wt% for ABT199 in comparison with boron‐containing NP (Table S4, Supporting Information), verifying that boronic ester bond and B—N coordination benefit drug loading. Thus, we further used the NP to encapsulate other catechol‐containing drugs including MIM1, epigallocatechin gallate (EGCG), and chlorogenic acid (CA). As expected, the three drugs could be robustly loaded in NP and exhibited decent DLE of 83.2%–95.6% at a theoretical DLC of 10 wt% (Table S5, Supporting Information). Of note, the superb loading level of different drugs in NPAT facilitate the adjustment of drug species and ratios in nanodrugs.

**Table 2:** Characterization of NPAT

Entry	TW37			ABT199			Size^b)^ [nm]	PDI^b)^	*ξ* ^c)^ [mV]
	DLC [wt%]		DLE^a)^ [%]	DLC [wt%]		DLE^a)^ [%]			
	Theo.	Determ.^a)^		Theo.	Determ.^a)^				
1	10.0	9.4	93.4	10.0	10.0	100	98	0.12	−1.1
2	10.0	9.3	93.1	14.3	14.2	99.3	103	0.06	−1.3
3	10.0	9.3	92.6	18.2	17.9	98.2	112	0.17	−2.2
4	10.0	9.3	92.1	25.0	24.5	97.6	125	0.05	−1.4
5	14.3	13.2	91.5	10.0	10.0	99.8	106	0.04	−1.2
6	18.2	16.7	90.6	10.0	9.9	98.5	115	0.05	−1.8
7	25.0	22.7	88.4	10.0	9.8	98.0	127	0.09	−2.1

^a)^ Determined by HPLC;

^b)^ Determined by DLS (1.0 mg mL^−1^, 25 °C);

^c)^ Determined by electrophoresis in PBS (1.0 mg mL^−1^, 25 °C).

In comparison with mono drug‐loaded nanosystems (NPA, NPT), NPAT exhibited slightly bigger sizes and the average diameter increased from 98 to 127 nm with elevating DLC (Table 2). Besides, NPA, NPT, and NPAT all revealed narrow size distribution (PDI < 0.2) and slightly negative surface charge of −2.2 to −1.1 mV. The size distribution and spherical morphology of NPAT were verified by transmission electron microscopy (TEM) images (Figure 1F). In sharp contrast with multiple distribution of blank NP measured by DLS, all drug‐loaded NP (NPA, NPT, NPAT) presented monodispersity and NPAT showed little change of size and size distribution against 100‐fold dilution, serum, and 30‐d storage at 4 °C (Figure 1H,I), corroborating that the interactions (boronic ester bond, *π*–*π* stacking, B—N coordination, etc.) between drugs and polypeptide segments benefit the stability of nanodrugs. Boronic ester bonds not only promote strong interactions of drugs with nanovehicles under physiological conditions, but also provide acid/H_2_O_2_‐triggered responsivity.^[^ ^19^, ^21^ ^]^ Indeed, NPAT revealed obvious dissociation and swelling after incubating in phosphate buffered salt (PBS) (pH 5.5) or PBS with 100 × 10^−6^ m H_2_O_2_ for 6 h (Figure 1J). Besides, enzymes like proteinase K (PK) could induce the nanoparticle swelling and aggregation as characterized by the presence of new bands at around 800 and 8000 nm owing to the enzymatic degradation of polypeptide segments. Similar enzyme‐responsive behavior has been observed in micelles formed from PEG‐PTyr copolypeptides and poly(l‐glutamic acid)‐*graft*‐PEG/combretastatin A4 prodrugs,^[^ ^22^ ^]^ which might be attributed to the cleavage of the connection part between PEG and polypeptide blocks, facilitating the removal of PEG shell, and nanoparticle dissociation and aggregation. Under shaking (200 rpm) at 37 °C, NPAT revealed swiftly trigged drug release against 50‐fold volume of release medium as a result from the cleavage of boronic ester and peptide bonds, in which around 66%, 80%, and 82% of drugs was released from nanoparticles within 48 h at pH 5.5, in the presence of H_2_O_2_ (100 × 10^−6^ m) and PK (12 units mL^−1^), respectively (Figure 1K and Figure S6, Supporting Information). On the contrary, less than 20% of drug leakage from NPAT was observed within 48 h in PBS at pH 7.4.

### 2.3 Hemocompatibility and Cytotoxicity

**Figure 2:**
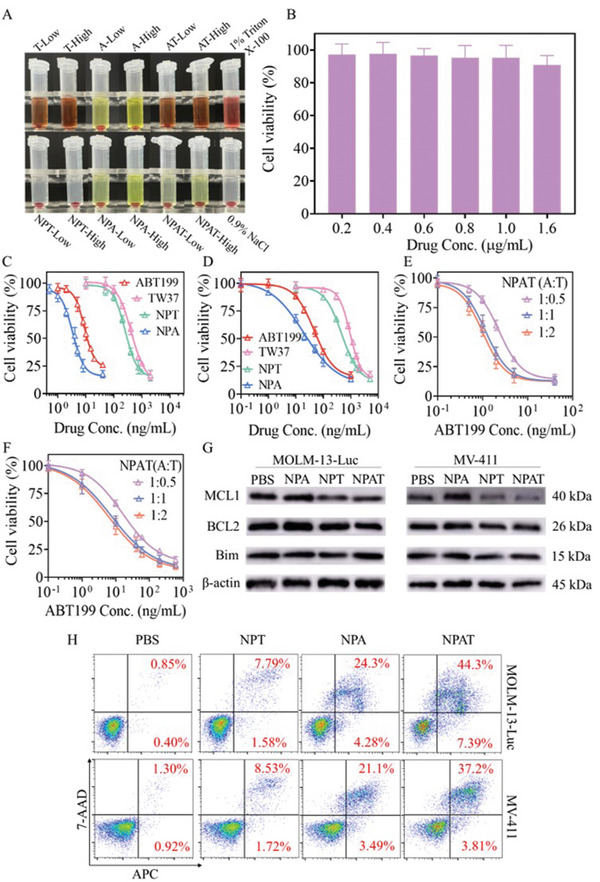
Hemocompatibility and cytotoxicity assay. A) Images of erythrocytes treated with free drugs and nanodrugs at low (100 µg mL^−1^) or high (200 µg mL^−1^) concentration. 1% Triton X‐100 and 0.9% NaCl were used as positive and negative controls, respectively. B) Cytotoxicity of NPAT at an A:T weight ratio of 1:1 in L929 cell following 48 h incubation (*n* = 6). Cell viability of MOLM‐13‐Luc C,E) and MV‐411 D,F) cells treated with different formulations for 48 h (*n* = 6). G,H) Protein expression and apoptosis of MOLM‐13‐Luc (ABT199: 10 ng mL^−1^, TW37: 10 ng mL^−1^) and MV‐411 (ABT199: 60 ng mL^−1^, TW37: 60 ng mL^−1^) cells treated with NPA, NPT, NPAT (A:T = 1:1) for 48 h. G) Protein expression determined by western blot analysis. H) Cell apoptosis assessed by annexin V‐APC/7‐AAD double staining technique.

Although free TW37 induced serious erythrocyte lysis with a hemolysis rate of over 55% at a low concentration of 100 µg mL^−1^, the introduction of NP remarkably improved the hemocompatibility, in which erythrocytes treated with NPT at a concentration up to 200 µg mL^−1^ revealed negligible cell lysis (**Figure** **2**A), providing TW37 with the potential of i.v. administration. Further supplement of ABT199 in NPT nanodrugs generated no additional hemolysis, and the dual drug‐loaded NPAT exhibited similar erythrocyte aggregates to negative control (0.9% NaCl), signifying their superb hemocompatibility. Both free ABT199 and NPA at different concentrations presented decent hemocompatibility with a hemolysis rate of lower than 5% (Figure S7, Supporting Information). The cytotoxicity of blank NP was evaluated by CCK8 assay with both cancer cells (MOLM‐13‐Luc and MV‐411 cells) and normal cells (L929 cells). The results demonstrated that all cells treated with NP at a concentration up to 200 µg mL^−1^ for 48 h presented a cell viability of over 90% (Figure S8, Supporting Information), verifying their satisfactory cytocompatibility. Besides, mice administered with NP at dosages of 10 and 20 mg kg^−1^ through intravenous injection displayed little body weight loss within 14 d (Figure S9, Supporting Information), which supported that NP had decent in vivo safety. More importantly, L929 cells following 48 h treatment with NPAT at drug concentrations up to 1.6 µg mL^−1^ revealed a cell viability of over 90% (Figure 2B), signifying the selective toxicity toward cancer cells of NPAT.

CCK8 assay revealed that NPT and NPA had higher cytotoxicity against MOLM‐13‐Luc and MV‐411 cells than corresponding free drug (Figure 2C,D). The enhanced cytotoxicity of nanodrugs might attribute to their different internalization pathway, controllable drug ratios, and improved retention in cancer cells, which has been verified by nanoparticulate paclitaxel, vincristine and DM1 in previous reports.^[^ ^23^ ^]^ Dual drug‐loaded NP further increased the cytotoxicity, in which NPAT at ABT199/TW37 (A:T) weight ratio of 1:1 displayed an IC_50_ of 1.15 ng mL^−1^ in MOLM‐13‐Luc cells that was around threefold and 222‐fold lower than NPA and NPT, respectively (Figure 2E). Further increasing the TW37 proportion to an A:T weight ratio of 1:2 afforded slightly lower IC_50_ for ABT199 while higher IC_50_ for TW37, and decreasing TW37 proportion (A:T = 1:0.5) generated obviously higher IC_50_ for ABT199 (Table S1, Supporting Information). Although NPAT generally afforded higher IC_50_ in MV‐411 cells than in MOLM‐13‐Luc cells, the nanodrugs revealed obvious synergetic toxicity with a CI of around 0.35 in both MV‐411 and MOLM‐13‐Luc cells at an A:T weight ratio of 1:1 (Figure 2F; Tables S1 and S2, Supporting Information).

### 2.4 Mitochondrial Membrane Permeability, Protein Expression, and Cell Apoptosis

The membrane permeability of mitochondria was investigated using JC‐1 fluorescence probe, and the results showed that all formulations revealed significantly decreased aggregation percentage (PE) and increased monomer percentage (FITC) of JC‐1 in comparison with PBS group (Figure S10A, Supporting Information), in which NPAT presented less than 2% of JC‐1 aggration/monomer ratios in both MV‐411 and MOLM‐13 cells (Figure S10B, Supporting Information), verifying that the nanodrugs could remarkably elevate the permeabilization of mitochondrial outer membrane. Western blot analysis revealed that BCL2 and MCL1 proteins were overexpressed in both MOLM‐13‐Luc and MV‐411 cells, and the formulations containing TW37 (NPT, NPAT) efficiently downregulated the MCL1 expression (Figure 2G). The anti‐apoptotic function of BCL2 protein can be neutralized by binding inhibitors like ABT199 in the hydrophobic groove,^[^ ^12^ ^]^ and ABT199 nanoformulations caused little change of BCL2 protein in both AML cells. Meanwhile, MOLM‐13‐Luc and MV‐411 cells treated with NPT, NPA, and NPAT all demonstrated noticeable activation of caspase‐9, and NPAT presented strongest activation effect (Figure S10C, Supporting Information). Annexin V‐APC/7‐AAD double staining technique demonstrated that NPAT, NPT, and NPA induced enhanced cell apoptosis in both MOLM‐13‐Luc and MV‐411cells in comparison with the corresponding free drugs (Figure 2H and Figure S11, Supporting Information). In MOLM‐13‐Luc cells, NPAT demonstrated the strongest pro‐apoptotic capacity and induced 44.3% of late apoptosis, which was around 1.8‐ and 5.7‐fold higher than that of NPA and NPT groups, respectively.

### 2.5 In Vivo Antitumor Efficacy

**Figure 3:**
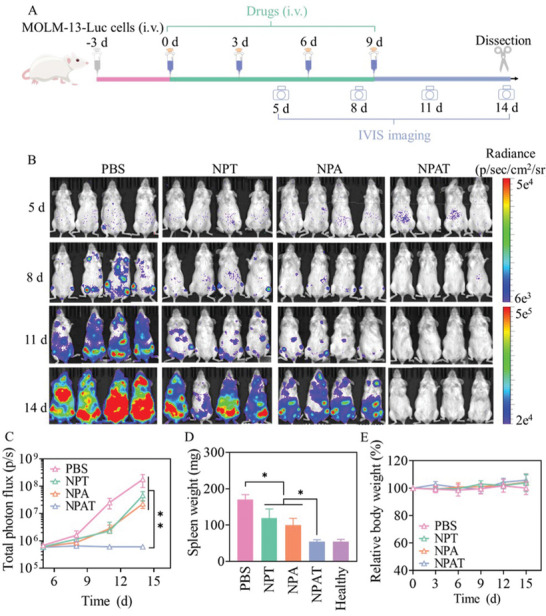
In vivo treatment of NPAT toward orthotopic MOLM‐13‐Luc AML in mice. A) Overview of the experimental scheme. The mice were i.v. injected with PBS, NPT, NPA, or NPAT on days 0, 3, 6, and 9, and leukemic infiltration was visualized by bioluminescence imaging on days 5, 8, 11, and 14. B) Bioluminescent images. C) Mean bioluminescence over time. D) Spleen weight on day 14. E) Body weight changes. Statistical analysis was performed by two‐tailed Student's *t*‐test for (C) and (D), *n* = 4, *p < 0.05, **p < 0.01.

The antitumor activity of NPAT was firstly examined in orthotopic MOLM‐13‐Luc cancer‐bearing B‐NDG mice in vivo (**Figure** **3**A). Mice in PBS group presented fast increase of bioluminescence derived from MOLM‐13‐Luc cancer cells (Figure 3B), and were featured with the paralysis of hind legs and whitish auricle. In contrast, NPA and NPT significantly retarded the infiltration of MOLM‐13‐Luc cells, especially in the initial 11 d, and NPAT induced nearly complete infiltration inhibition of MOLM‐13‐Luc cells without visible bioluminescence signal even at the end of the experimental period (Figure 3B,C). On day 14, blood was collected from the abdominal cavity of mice for blood routine test and biochemical analysis. Comparing with significantly elevated white blood cells, and decreased red blood cells and platelets in PBS group, NPAT group presented equivalent level of blood cells and platelets to healthy mice (Figure S12, Supporting Information), indicating that NPAT might inhibit leukemia cell infiltration and restore hematopoietic function. Consistently, mice treated with NPAT revealed similar spleen weight to healthy mice, in sharp contrast with massive splenomegaly in PBS, NPA, and NPT groups (Figure 3D). Of note, mice treated with all nanoformulations (NPA, NPT, and NPAT) exhibited similar level of alanine aminotransferase (ALT), aspartate aminotransferase (AST), and urea (Figure S13, Supporting Information) as well as little body weight change (Figure 3E), signifying the notable safety of nanodrugs.

**Figure 4:**
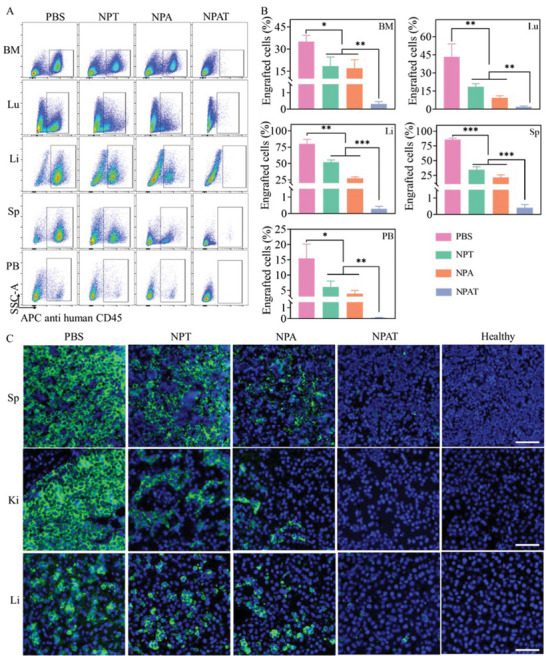
Invasion analysis of MOLM‐13‐Luc leukemia cells in main organs on day 14. A) Cell invasion in bone marrow (BM), lung (Lu), liver (Li), spleen (Sp), and peripheral blood (PB) determined by flow cytometry. B) Quantitative analysis of invaded leukemia cells. Statistical analysis was performed by two‐tailed Student's *t*‐test, *n* = 3, *p < 0.05,**p < 0.01, ***p < 0.001. C) Immunohistochemical staining of histologic sections using human CD45 antibody. Scale bar, 50 µm.

The infiltration of leukemia cells extracted from different organs were labeled with APC‐anti‐human‐CD45 antibody and determined by flow cytometry. PBS group showed massive invasion of MLOM‐13‐Luc leukemia cells, in which the proportion of leukemia cells in bone marrow (BM), lung (Lu), liver (Li), spleen (Sp), and peripheral blood (PB) was 35.2%, 39.9%, 80.5%, 86.0%, and 15.4%, respectively (**Figure** **4**A,B). In contrast, NPA, NPT, and NPAT significantly inhibited the infiltration of leukemia cells in various organs, and NPAT revealed best inhibition ability with minimum leukemia invasion in BM (<0.4%) and all main organs (<2%). Although NPT and NPA groups obviously reduced the leukemia infiltration, the engrafted leukemia cells was over 15% in bone marrow, liver, and spleen on day 14, verifying the synergistic efficacy of dual‐drug combination. Immunohistochemical analysis using human CD45 and hematoxylin and eosin (H&E) staining further demonstrated that NPAT almost completely eliminated the infiltration of leukemia cells in main organs, while mice in PBS group revealed dense leukemia cells in varying organs (Figure 4C and Figure S14, Supporting Information).

**Figure 5:**
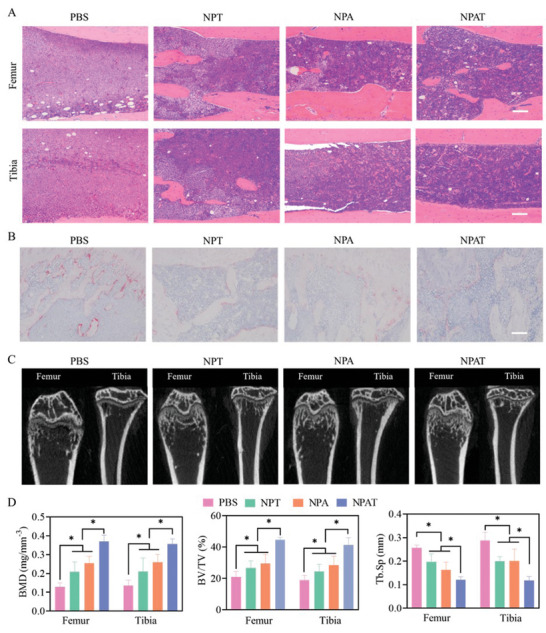
Analysis of femur and tibia of orthotopic MOLM‐13‐Luc AML bearing mice in different treatment groups. A) H&E staining, scale bar: 100 µm. B) Osteoclast identification by TRAP staining, scale bar: 100 µm. C) Micro‐CT images. D) Quantitative analysis of bone mineral density (BMD), bone volume/tissue volume (BV/TV), and trabecular separation/spacing (Tb.Sp). Statistical analysis was performed by two‐tailed Student's *t*‐test, *n* = 3, *p < 0.05.

As characterized by H&E staining of femur and tibia of hind legs, massive leukemia cells and a small number of hematopoietic cells distributed in the bone marrow cavity in PBS group, while NPA, NPT, and NPAT remarkably decreased the invasion of MOLM‐13‐Luc leukemia cells (**Figure** **5**A). Of note, mice treated with NPAT presented little leukemia cells and similar level of hematopoietic cells in bone marrow to healthy mice (Figure S15, Supporting Information), in consistent with normalized blood cells and platelet measured by blood routine analysis. Moreover, osteolytic lesions induced by the disruption of the balance between osteoclasts and osteoblasts often cause osteoporosis and osteolysis.^[^ ^24^ ^]^ Tartrate‐resistant acid phosphatase (TRAP) is recognized as one of the main markers of osteoclasts.^[^ ^23b^ ^]^ Figure 5B demonstrated that plentiful osteoclasts were enriched in PBS group, while NPA, NPT, and NPAT induced significantly decreased osteoclasts with an equivalent level to healthy mice. Micro‐CT images showed mice treated with PBS had lowest bone mineral density (BMD) in both femur and tibia, and NPA, NPT as well as NPAT greatly elevated the BMD, in which NPAT group presented around 2.8 times higher BMD than PBS (Figure 5C,D). Comparing with PBS group, NPAT induced significantly increased bone mass with around two times higher bone surface/tissue volume (BS/TV) and bone volume/tissue volume (BV/TV). Mice treated with PBS revealed increased trabecular spacing (Tb.Sp), decreased trabecular thickness (Tb.Th) and trabecular number (Tb.N), signifying obvious bone damage as a result from osteolytic lesions induced by AML. NPAT induced normally spatial morphology and structure of bone trabeculas with similar level of Tb.Sp, Tb.Th, and Tb.N to healthy mice (Figure 5D and Figure S16, Supporting Information).

**Figure 6:**
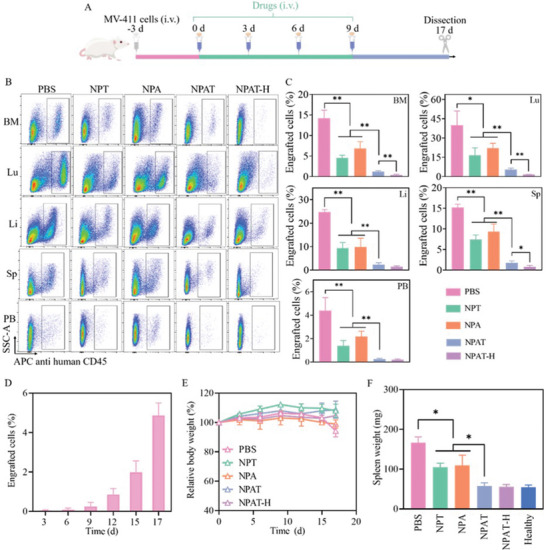
In vivo anticancer effect of NPAT toward orthotopic MV‐411 AML in mice. The mice were i.v. injected with PBS, NPT, NPA, or NPAT on days 0, 3, 6, and 9, respectively. A) Overview of the experimental scheme. B) Infiltration of MV‐411 cells in BM, Lu, Li, Sp, and PB. C) Quantitative analysis of cell infiltration (*n* = 3). D) Change of engrafted MV‐411 cells in PB of mice with time in PBS group (*n* = 4). E) Body weight changes of mice (*n* = 4). F) Spleen weights of mice following 17‐d treatment with different formulations (*n* = 4). Statistical analysis for (C) and (F) was performed by two‐tailed Student's *t*‐test, *p < 0.05, **p < 0.01.

The therapeutic efficacy of NPAT was further evaluated in orthotopic MV‐411 tumor‐bearing B‐NDG mice in vivo (**Figure** **6**A). PBS group showed massive invasion of MV‐411 leukemia cells with around 14.2%, 40.0%, 24.7%, 15.0%, and 4.9% leukemia cells in BM, Lu, Li, Sp, and PB on day 17, respectively (Figure 6B,C). In contrast, NPA, NPT, and NPAT significantly inhibited the infiltration of leukemia cells in main organs, and NPAT revealed best inhibition ability with minimum leukemia invasion in BM (<2%), verifying the synergistic efficacy of NPAT. Consistently, mice treated with NPAT displayed significant survival advantages with an extended median survival time of 28 d, which was 11 d longer than PBS group (Figure S17, Supporting Information). In contrast, NPA and NPT groups revealed 2–3 d longer median survival time than PBS group. Increasing the drug dosages from 10 to 15 mg kg^−1^ for both ABT199 and TW37 (NPAT‐H) afforded improved therapeutic outcomes, and revealed better inhibition ability with around 3.4‐fold less leukemia invasion in BM (0.36% vs 1.21%). Besides, NPAT‐H afforded around 1.9‐ and 3.4‐fold less engrafted MV‐411 cells than NPAT in spleen and lung, respectively. Figure 6D revealed that mice treated with PBS had swift increase of leukemia cells in peripheral blood with time, which combined with obvious hind leg paralysis and whitish auricle suggested that the successful establishment and rapid progression of MV‐411 leukemia model. In sharp contrast with massive splenomegaly in PBS, NPA, and NPT groups, mice treated with NPAT revealed similar spleen weight to healthy mice (Figure 6E). Importantly, NPA, NPT, and NPAT induced little body weight change during treatment, while mice in PBS group exhibited around 7% weight loss mainly attributed to the invasion of leukemia cells (Figure 6F).

## 3 Conclusion

We demonstrated that nanovehicles developed from phenylboronic acid‐functionalized polypeptides could enable nearly quantitative coencapsulation and triggered release of both BCL2 and MCL1 inhibitors for synergetic and potent therapy of AML. Leveraging the dynamic boronic ester bonds, B—N coordination, and *π*–*π* stacking formed between drugs and polypeptide segments, the dual inhibitor‐loaded nanoparticles (NPAT) revealed remarkable stability, adjustable drug ratios, acid/enzyme/H_2_O_2_‐responsive drug release, and significant cytotoxicity toward MOLM‐13‐Luc and MV‐411 AML cells with low IC_50_ of 1.15 and 7.45 ng mL^−1^ at an ABT199/TW37 weight ratio of 1:1, respectively. Besides, NPAT demonstrated significant inhibition of tumor cell infiltration in bone marrow, lung, liver, spleen, and peripheral blood, potent suppression of tumor growth, and remarkably elevated mouse survival in both MOLM‐13‐Luc and MV‐411 AML models. Thus, the phenylboronic acid‐functionalized smart nanomedicines featured with facile construction, adjustable drug combination, superior safety, synergetic and potent therapeutic outcomes can be easily extended to treat different cancers.

## 4 Experimental Section

### Synthesis and Characterization of PEG‐*b*‐P(BPA‐*co*‐Tyr)

PEG‐NH_2_ (*M*_n_ = 5.0 kg mol^−1^) was employed as an initiator to copolymerize l‐boronophenylalanine *N*‐carboxyanhydride (BPA‐NCA) and l‐tyrosine *N*‐carboxyanhydride (Tyr‐NCA) monomers. Under a nitrogen atmosphere, to a solution of PEG‐NH_2_ (350 mg, 0.07 mmol) in DMF (3.5 mL) was rapidly added a mixture of BPA‐NCA (430 mg, 1.83 mmol) and Tyr‐NCA (111 mg, 0.57 mmol) in DMF (3.5 mL). The reaction proceeded at 80 °C for 72 h, considering that the polymerization of BPA‐NCA is not controllable and the polymerization efficiency was rather low at lower temperature (Figure S2B, Supporting Information). Following the precipitation in an excess of diethyl ether, the raw product was further purified by redissolving in methyl alcohol and precipitating in diethyl ether thrice. Yield: 83%. ^1^H NMR (400 MHz, DMSO*‐d*_6_/CD_3_OD*‐d*_4_, 5:1, v/v (Figure S2, Supporting Information), *δ*): 7.68 and 7.22 (—C_6_ *H_4_* B(OH)_2_), 6.95 and 6.61 (—C_6_ *H_4_* OH), 4.47 and 4.37 (—COC*H*NH—), 3.52 (—OC*H_2_* C*H_2_* O—), 2.82 and 2.63 (—C_6_H_4_C*H_2_*—).

### Preparation and Characterization of NPAT

TW37 and ABT199 were readily loaded into nanoparticles via a solvent‐exchange method. Briefly, the mixed solution consisting of PEG‐*b*‐P(BPA‐*co*‐Tyr) (20 mg mL^−1^, 50 µL), ABT199 (20 mg mL^−1^, 5.6 µL), and TW37 (20 mg mL^−1^, 5.6 µL) was dropwise added to HEPES buffer (pH 7.4, 10.0 × 10^−3^ m, 940 µL) under stirring at room temperature. Thus formed NPAT was sequentially dialyzed (MWCO 3500 Da) against HEPES and PBS for 6 h to remove free drugs. The dialysis medium was replaced every hour. Dialysis against aqueous solution for 6 h was selected to remove free drugs from NPAT considering that the extension of dialysis period to 10 h afforded similar drug loading efficiency to 6 h dialysis. TW37‐loaded nanoparticles (NPT) and ABT‐199‐loaded nanoparticles (NPA) were similarly fabricated except that one free drug was employed. The colloidal stability of nanodrugs under different conditions was monitored by measuring the size change using DLS. Drugs were quantified using high performance liquid chromatography (HPLC), and the DLC and DLE were calculated according to the following formulas

(1) $$\begin{array}{ccc} \mathrm{DLC}\;(\mathrm{wt}\%) & = & (\mathrm{weight}\;\mathrm{of}\;\mathrm{loaded}\;\mathrm{drug}/\\ & & (\mathrm{weight}\;\mathrm{of}\;\mathrm{polymer}+\mathrm{weight}\;\mathrm{of}\;\mathrm{loaded}\;\mathrm{drug}))\times 100 \end{array}$$

(2) DLE(%)=(weightofloadeddrug/weightoffeddrug)×100

### Hemolysis Assay and Cytotoxicity

The blood of healthy mice was collected in anticoagulant vessels and centrifuged (3000 RPM, 4 °C, 10 min) to remove the upper serum. The residue solution was washed with saline solution (0.9% NaCl) for several times until the supernatant was colorless. Then, the obtained red blood cells were dispersed in saline solution (0.9% NaCl) to acquire 2% (v/v) of cell suspension. After mixing with an equal volume of TW37, ABT199, TW37/ABT199, NPT, NPA, or NPAT at low (100 µg mL^−1^) or high (200 µg mL^−1^) concentration, the solution was incubated at 37 °C for 20 min. Following the removal of remained red blood cells through centrifugation, the absorbance of supernatant was determined by ultraviolet spectrophotometer at 570 nm. 0.9% NaCl saline and 1% Triton X‐100 solution were employed as negative and positive controls, respectively. Hemolysis rate (HR) was calculated with the following formula: HR (%) = (*A*_S_ − *A*_NC_)/(*A*_PC_ − *A*_NC_) × 100. *A*_S_, *A*_NC_, and *A*_PC_ represent ultraviolet absorbance of samples, negative control, and positive control, respectively.

The toxicity of NPAT to MOLM‐13‐Luc and MV‐411 cells was assessed by CCK8 assay. The cells cultured in a 96‐well plate (2 × 10^4^ cells/well) with 1640 medium were treated with TW37, ABT199, TW37/ABT199, NPT, NPA, and NPAT at varying concentrations and drug ratios for 48 h. Then, 10 µL of CCK8 solution was added, and the cells were incubated for another 3 h. The absorbance of the above solution was acquired by a microplate reader at 450 nm, and the cell viability was determined by comparing the absorbance of different samples with average absorbance of PBS group. The combination index (CI) was calculated according to Chou and Talalay's method^[^ ^25^ ^]^

(3) $$\mathrm{CI}=\frac{C_{A}}{S_{A}}+\frac{C_{T}}{S_{T}}$$

where *S*_A_ and *S*_T_ represent the IC_50_ values of NPA and NPT, respectively. *C*_A_ and *C*_T_ are the IC_50_ values of ABT199 and TW37 in NPAT, respectively. CI < 1, CI = 1, and CI > 1 indicate synergistic, additive, and antagonistic effects, respectively.

### Protein Expression and Cell Apoptosis

The expression of MCL1 and BCL2 proteins in MOLM‐13‐Luc and MV‐411 cells was investigated by western blot experiments. MOLM‐13‐Luc cells (2 × 10^5^ cells/well) were treated with 200 µL of NPT, NPA, or NPAT (TW37: 10 ng mL^−1^, ABT: 10 ng mL^−1^) for 48 h. After washing for three times, the cells were lysed for 20 min and centrifuged to obtain the supernatant. The proteins in the supernatant were quantified using BCA protein assay kit, and then boiled with bromophenol blue solution for 5 min. After separating by electrophoresis, different proteins were transferred to a polyvinylidene fluoride membrane. After blocking with 5% skimmed milk for 1 h at room temperature, the protein bands were treated with rabbit BCL2, MCL1, Bim, cleaved caspase‐9, and *β*‐actin antibody (1000‐fold dilution) overnight at 4 °C, respectively, followed by incubating with goat anti‐rabbit antibody (3000‐fold dilution) for another 1.5 h. Proteins were observed using a chemiluminescence detection system (Amersham Imager 600, General Electric Company).

For the evaluation of cell apoptosis, MOLM‐13‐Luc cells (2 × 10^5^ cells/well) seeded in 12‐well plates were treated with different drugs (ABT199: 10 ng mL^−1^, TW37: 10 ng mL^−1^) for 48 h. Following the addition of binding buffer, the collected cells were stained with Annexin V‐allophycocyanin (APC) and 7‐amino‐actinomycin D (7‐AAD) at room temperature in darkness for 5 min. A total of 20 000 cells were analyzed per sample. The early and late apoptotic cells were determined using flow cytometry within 1 h, and analyzed using FlowJo V10 software. The protein expression and cell apoptosis of MV‐411 were similar evaluated except that higher drug concentrations (ABT199: 60 ng mL^−1^, TW37: 60 ng mL^−1^) were employed.

### In Vivo Antitumor Performance

All animal procedures were carried out in accordance with the protocols approved by Laboratory Animal Center and Animal Care and Use Committee of Soochow University. AML mouse model was established by intravenous transplantation of MOLM‐13‐Luc or MV‐411 cells (5 × 10^5^ cells per mouse) into B‐NDG mice (female, six weeks, 18–22 g). MOLM‐13‐Luc tumor‐bearing mice were randomly divided and administrated with different formulations (PBS, NPT, NPA, NPAT) on the third day after inoculation. The day on which the drugs were first given was designated as day 0, and all formulations were intravenously injected on days 0, 3, 6, and 9 (TW37: 10 mg kg^−1^, ABT199: 10 mg kg^−1^). Bioluminescence derived from MOLM‐13‐Luc cells were acquired following i.p. injection of d‐luciferin (1.5 mg/mouse) on day 5, 8, 11, and 14 using interactive video information system (IVIS Lumina III, PerkinElmer, USA) to monitor the tumor growth profiles. The bioluminescence intensity of the mice were relative to their values on day 5. On day 14, PB was collected from eye socket and stored in PBS (containing 1% v/v FBS). Besides, celiac artery blood was harvested during dissection for blood routine and biochemistry measurements. Then, the mice were sacrificed, and lung, liver, spleen, and one hind leg were milled to extract cells. The cells collected from PB and main organs were resuspended in PBS (containing 1% v/v FBS), and then treated with red blood cell lysis buffer solution for 15 min to lyse red blood cells. The leukemia cells were stained with anti‐CD45‐APC antibody for 20 min and quantified using FlowJo V10 software. A total of 20 000 cells were analyzed per sample. The infiltration of leukemia cells in main organs and BM was further assessed by H&E staining, and osteoclasts in tibias and femurs were explored by TRAP staining. Micro‐CT measurement was used to evaluate the BMD, bone mass, morphology, and structure of femur and tibia. Differently, the tumor progression of MV‐411 leukemia treated with different formulations was tracked by measuring PB collected from the eye socket during the experimental period (days 3, 6, 9, 12, 15, and 17). The MV‐411 leukemia‐bearing mice were sacrificed on day 17 to evaluate the cell infiltration in BM and main organs. The spleen of mice was weighed at the end of experiment and the weight of tumor‐bearing mice was measured every 3 d. The body weight of the mice was relative to their values on day 0.

### Statistical Analysis

Data was represented as means ± standard deviation (SD). The significant differences among groups were determined using GraphPad Prism 8 by two‐tailed Student's *t*‐test or one‐way ANOVA or log‐rank Mantel–Cox test. For bioinformatics analysis, the statistical significances were assessed by the one‐way ANOVA. For survival analysis, using the log‐rank (Mantel–Cox) test was applied. For others, statistical differences between groups were assessed by two‐tailed Student's *t*‐test. Statistical significance was defined as *p < 0.05, **p < 0.01, ***p < 0.001.

## Conflict of Interest

The authors declare no conflict of interest.

## Supporting information

Supporting Information

## Data Availability

The data that support the findings of this study are available from the corresponding author upon reasonable request.
