# Supplementary material for: Codelivery of BCL2 and MCL1 Inhibitors Enabled by Phenylboronic Acid‐Functionalized Polypeptide Nanovehicles for Synergetic and Potent Therapy of Acute Myeloid Leukemia
Source: Adv Sci (Weinh). 2023 Jan 22;10(8):2204866. doi: 10.1002/advs.202204866 (PMC10015845; doi:10.1002/advs.202204866)
Supplement: Supplementary file 1 — Supporting Information [file ADVS-10-2204866-s001.pdf]

Supporting Information

Codelivery of BCL2 and MCL1 Inhibitors Enabled by Phenylboronic Acid-Functionalized Polypeptide Nanovehicles for Synergetic and Potent Therapy of Acute Myeloid Leukemia

Jiguo Xie[#], Xiaofei Zhao[#], Peng Zhang, Yueyue Zhang, Ru Cheng, Zhiyuan Zhong, and Chao Deng**

Biomedical Polymers Laboratory, and Jiangsu Key Laboratory of Advanced Functional Polymer Design and Application, College of Chemistry, Chemical Engineering and Materials Science, and State Key Laboratory of Radiation Medicine and Protection, Soochow University, Suzhou 215123, China.

Materials

Tyrosine (Tyr, Gill Biochemical Shanghai Co., Ltd.), L-4-dihydroxyboronphenylalanine (BPA, Beijing Mreda Technology Co., Ltd.), *α*-methoxy-*ω*-amino-poly(ethylene glycol) (PEG-NH_2, M_n = 5.0 kg/mol, Xiamen Sinopeg Biotechnology Co., Ltd.) were used as received. Triphosgene (BTC, Shanghai Aladdin Biochemical Technology Co., Ltd.) was recrystallized with ethyl acetate before use. Tetrahydrofuran (THF) and petroleum ether were purified using a solvent purification system (Innovative Technology, USA) before use. TW37, ABT199, MIM1, EGCG and chlorogenic acid (CA) were purchased from MedChemExpress (Shanghai, China). MCL1 antibody, BCL2 antibody and Bim antibody (Beyotime), β-actin antibody and cleaved caspase-9 antibody (CST) were used according to the manufacturer's instructions. Human APC- antibody specific to human CD45 (anti-CD45-APC, BD Biosciences) and BCA assay kit (Thermo Scientific) were used according to the manufacturer's instructions. JC-1 mitochondrial membrane potential assay kit (Solarbio) were used following the manufacturer's instructions. *N*, *N*-Dimethylformamide (DMF) was dried with $MgSO_4$ and distilled under reduced pressure before use. L-4-dihydroxyboronphenylalanine-*N*-carboxylactone (BPA-NCA) and L-tyrosine *N*-carboxyanhydride (Tyr-NCA) were prepared according to previous literatures.[1] All other reagents and solvents were purchased from Sinopharm Chemical Reagent Co. Ltd. and used as received.

Characterization

^{1}H, ^{11}B, and 2D ^{1}H-^{1}H NOESY nuclear magnetic resonance (NMR) spectra were recorded on a Unity Inova 400 spectrometer operating at 400 MHz. $BF_3·OEt_2$ was used as the external reference to calibrate the ^{11}B NMR spectra. The chemical shifts were calibrated against solvent signals. The molecular weight of the copolymers was measured by MALDI-TOF using trans-2-(3-(4-tertbutylphenyl)-2-methyl-2-propenylidene) malononitrile (DCTB)/CF_3COONa^+ (9/1, v/v) as a matrix. The polydispersity index was determined by a Waters 1515 gel permeation chromatograph (GPC) instrument equipped with two linear PL gel columns (500 A and Mixed-

C) following a guard column and a differential refractive-index detector. The measurements were performed using DMF (containing 10 mM LiBr) as the eluent at a flow rate of 1.0 mL/min at 30 ºC and a series of narrow polystyrene standards for the calibration of the columns. The sizes of nanoparticles were determined using dynamic light scattering (DLS). Measurements were carried out at 25 ºC by Zetasizer Nano-ZS from Malvern Instruments equipped with 633 nm He-Ne laser using backscattering detection. The zeta potential of nanoparticles was determined with Zetasizer Nano-ZS from Malvern Instruments equipped with a standard capillary electrophoresis cell. The drug concentrations of TW37 and ABT199 were detected by high performance liquid chromatograph (HPLC). The column temperature was set at 30 ºC, the flow rate was 1.0 mL/min, the injection volume was 10 μL, the UV detection wavelength was 300 nm, and the mobile phase was acetonitrile/ water (80/20, v/v).

Cell Culture and Animal Study

MOLM-13-Luc and MV-411 cell lines were purchased from the Chinese Academy of Sciences (Shanghai) Cell Bank. Cell culture dishes and 96-well plates were purchased from Thermo Fisher (Shanghai) Technology Co., Ltd. CCK8 was purchased from Sigma-Aldrich (Shanghai) Trading Co., Ltd. L929 cells, MOLM-13-Luc and MV-411 were cultured in 1640 medium containing penicillin (10 U/mL), streptomycin (0.1 mg/mL) and FBS (10%, v/v).

6-week female B-NDG (NOD.CB17-*Prkdc*^{scid}*Il2rg*^{tm1}/Bcgen) mice (body weight 19-22 g) were purchased from Biocytogen Pharmaceuticals Co., Ltd, housed under programmed light/dark conditions at 25 ºC and 55% humidity, and allowed free access to sterile food and water.

Alizarin Red S Competitive Binding Experiment

In order to verify that the boron ester bonds formed between PEG-*b*-P(BPA-*co*-Tyr) and TW37, alizarin red S (ARS) competitive binding experiment was performed. ARS with no fluorescence can combine with boronic acid moieties to form ARS complexes that often exhibit strong fluorescence. The fluorescence intensity of formed ARS complexes would decrease

through competitive binding with substances containing cis-1,2- or 1,3-diols. Briefly, a PEG-*b*-P(BPA-*co*-Tyr) solution (95.2 μg/mL, 0.5 mL) was thoroughly mixed with ARS solution (68.4 μg/mL, 0.5 mL) for 3 min. Then, predetermined amount (0, 1.44 μL, 2.87 μL, 4.31 μL, 5.74 μL, 14.4 μL, 28.7 μL) of TW37 (20 mg/mL) dissolved in DMSO was added to the mixed solution. After stirring with a shaker (37 ºC, 100 rpm) for 10 min, the fluorescence intensity at 520-720 nm was detected using a fluorophotometer with an excitation wavelength at 470 nm.

***In Vitro* Triggered Drug Release**

In vitro drug release was carried out in four different media (25 mL): (i) PBS (150 mM, pH 7.4), (ii) PBS (150 mM, pH 5.5), (iii) PBS (150 mM, pH 7.4) containing proteinase K (PK, 12 U/mL), and (iv) PBS (150 mM, pH 7.4) containing 100 μM H_2O_2. NPAT (0.5 mL, 0.5 mg/mL) added in a dialysis tube (MWCO 30 kDa) was immersed in different release media under shaking at 37 °C. At predetermined time points, 5.0 mL of release medium was withdrawn and refilled with fresh medium. The amount of TW37 and ABT199 in release medium was quantified using high performance liquid chromatography (HPLC). The cumulative drug release was calculated according to the following formula:

$$E_r = \frac{V_e \sum_1^{n-1} C_i + V_0 C_n}{m_{drug}}$$

Er: cumulative release of TW37 or ABT199 (%); *Ve*: displacement volume of release medium (5.0 mL); V_0: total volume of release medium (25 mL); C_i: release medium at the i-th sampling concentrations of TW37 or ABT199 (μg/mL); m_{drug}: total amount of TW37 or ABT199 in NPAT used for release (μg); n: number of replacement medium.

Mitochondrial Membrane Permeability

To analyze the mitochondrial membrane permeability of MOLM-13-Luc and MV-411 cells, the cells in 6-well plate (5 × 10^5 cells/well) were incubated with NPT, NPA or NPAT for 48 h. The drug concentration was 10 ng/mL for MOLM-13-Luc cells and 60 ng/mL for MV-411 cells. After removing the culture media, the cells were treated with JC-1 at 37 ºC for 20

min according to the user's manual of mitochondrial membrane potential assay kit. Then, the cells were subjected to flow cytometry analysis, and the ratios of the intact mitochondria to damaged mitochondria as compared to that of PBS group were calculated.

Immunohistochemical Analysis

The tissue was formalin-fixed, paraffin-embedded, sectioned (5 μm thickness), and mounted onto microscope slides. After deparaffinizing and rehydrating, the tissue sections were sequentially incubated with primary antibody against human CD45 (Abcam, UK) and biotinylated secondary antibody (ZSGB-Bio, China). Tyramide signal amplification (TSA) technology was used for fluorescence color rendering. Images were taken using a fluorescence microscope system under the same magnification.

References

[1] a) X. Gu, M. Qiu, H. Sun, J. Zhang, L. Cheng, C. Deng, Z. Zhong, *Biomater. Sci.* **2018**, *6*, 1526; b) Q. Zhang, Y. Liu, Y. Fei, J. Xie, X. Zhao, Z. Zhong, C. Deng, *Biomacromolecules* **2022**, *23*, 2989.

Table S1. IC_{50} and CI values of different formulations in MOLM-13-Luc cells.

Entry	Drug	IC_{50} (ng/mL)		CI
		ABT199	TW37	
1	ABT199	8.89	-	-
2	TW37	-	430.6	-
3	Free (A:T) = 1:0.5	6.30	3.15	0.72
4	Free (A:T) =1:1	5.08	5.08	0.58
5	Free (A:T) =1:2	4.98	9.96	0.58
6	NPA	3.37	-	-
7	NPT	-	254.9	-
8	NP (A:T) = 1:0.5	2.30	1.15	0.69
9	NP (A:T) = 1:1	1.15	1.15	0.35
10	NP (A:T) = 1:2	1.01	2.02	0.31

Table S2. IC_{50} and CI values of different formulations in MV-411 cells.

Entry	Drug	IC_{50} (ng/mL)		CI
		ABT199	TW37	
1	ABT199	50.50	-	-
2	TW37	-	928.8	-
3	Free (A:T) = 1:0.5	39.97	19.99	0.81
4	Free (A:T) =1:1	19.85	19.85	0.41
5	Free (A:T) =1:2	14.75	29.5	0.32
6	NPA	21.54	-	-
7	NPT	-	468.1	-
8	NP (A:T) = 1:0.5	16.10	8.05	0.76
9	NP (A:T) = 1:1	7.45	7.45	0.36
10	NP (A:T) = 1:2	6.29	12.58	0.32

Table S3. Characterization of NPT and NPA.

Entry	Drug	DLC (wt.%)		DLE [a]	Size[b]	PDI[b]	ξ[c]
		Theo. (wt.%)	Determ. (wt.%) [a]	(%)	(nm)		(mV)
1	TW37	10.0	9.9	98.8	79	0.19	-0.4
2		20.0	19.0	94.1	84	0.11	-1.2
3		30.0	28.7	93.7	87	0.11	-1.1
4		40.0	38.2	92.8	92	0.07	-1.6
5		50.0	48.0	92.2	99	0.12	-1.9
6	ABT199	10.0	10.0	100.0	80	0.16	-1.1
7		20.0	20.0	100.0	86	0.15	-0.9
8		30.0	29.7	98.3	109	0.19	-2.1
9		40.0	38.8	94.9	132	0.14	-1.3
10		50.0	48.6	94.5	148	0.12	-1.8

[a]Determined by HPLC. [b]Determined by DLS (PBS 7.4, 1.0 mg/mL, 25 °C). [c]Determined by electrophoresis (PBS 7.4, 1.0 mg/mL, 25 °C).

Table S4. Characterization of NPAT-BF

Entry	TW37			ABT199			Size[b]	PDI[b]	ξ[c]
	DLC (wt.%)		DLE [a]	DLC (wt.%)		DLE [a]	(nm)		(mV)
	Theo.	Determ.[a]	(%)	Theo.	Determ.[a]	(%)			
1	10	1.7	16.0	10	9.5	95.1	69	0.12	-3.3
2	10	1.8	16.4	18.2	16.4	88.4	77	0.12	-2.7
3	10	1.7	15.6	25.0	22.2	85.7	84	0.10	-1.6
4	18.2	1.7	7.7	10	9.5	94.3	72	0.11	-1.8
5	25.0	1.8	5.4	10	9.4	93.2	74	0.14	-2.1

[a]Determined by HPLC. [b]Determined by DLS (1.0 mg/mL, 25 °C). [c]Determined by electrophoresis in PBS (1.0 mg/mL, 25 °C).

Table S5. Characterization of nanoparticles loaded with MIM1, EGCG, and CA.

Entry	Drug	DLC (wt.%) Theo. (wt.%)	DLC (wt.%) Determ. (wt.%) [a]	DLE [a] (%)	Size[b] (nm)	PDI[b]	ξ[c] (mV)
1	MIM1	10.0	9.6	95.6	76	0.09	-2.6
2		20.0	18.9	93.1	83	0.11	-1.2
3	EGCG	10.0	8.7	86.3	72	0.13	-3.1
4		20.0	16.6	80.3	79	0.08	-2.2
5	CA	10.0	8.5	83.2	73	0.14	-0.9
6		20.0	16.5	78.9	77	0.10	-3.4

[a]Determined by HPLC. [b]Determined by DLS (1.0 mg/mL, 25 °C). [c]Determined by electrophoresis in PBS (1.0 mg/mL, 25 °C).

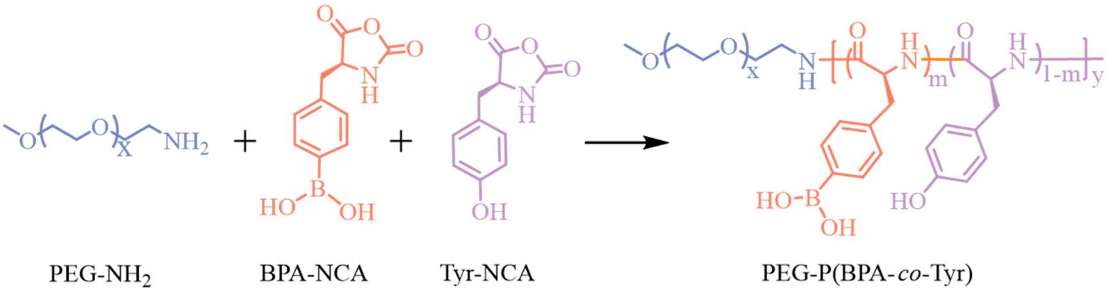

Scheme S1. Synthesis of PEG-*b*-P(BPA-*co*-Tyr). Condition: DMF, N_2, 80 °C, 3 d.

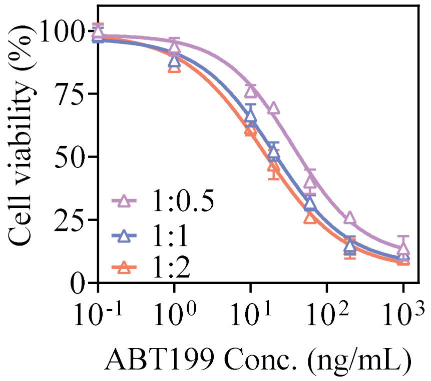

Figure S1. Cytotoxicity of MV-411 cells following the treatment with dual drugs at different ABT199/TW37 weight ratios of 1:0.5, 1:1 and 1:2 for 48 h (n = 6).

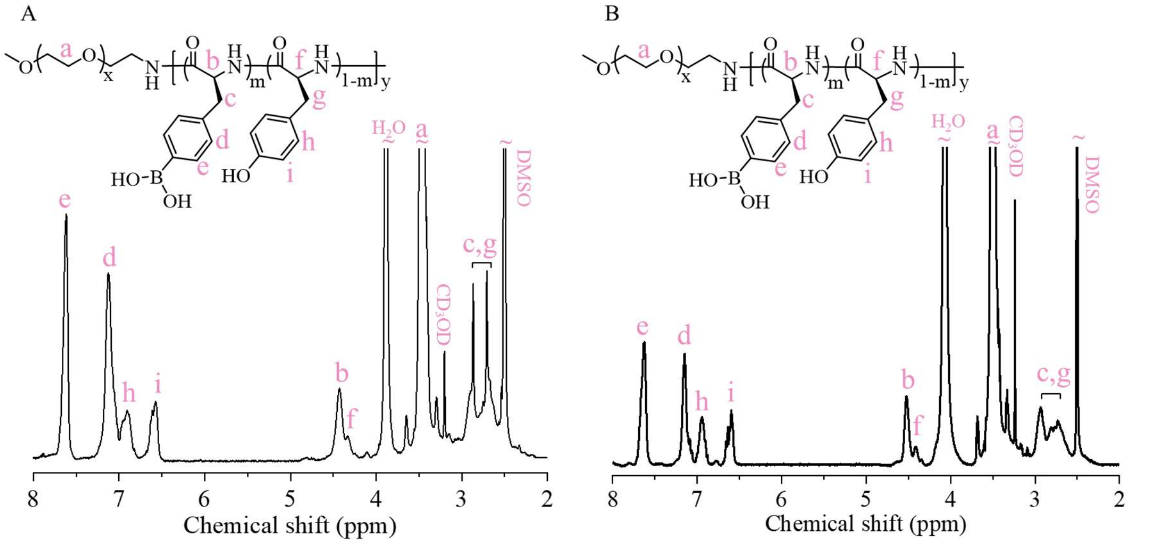

Figure S2. ^{1}H NMR spectra (400 MHz, DMSO-d_6/CD_3OD-d_4, 5:1, v/v) of PEG-*b*-P(BPA-*co*-Tyr). (A) Polymerization at 80 ºC for 3 d (Table 1, entry 1). (B) Polymerization at 60 ºC for 3 d.

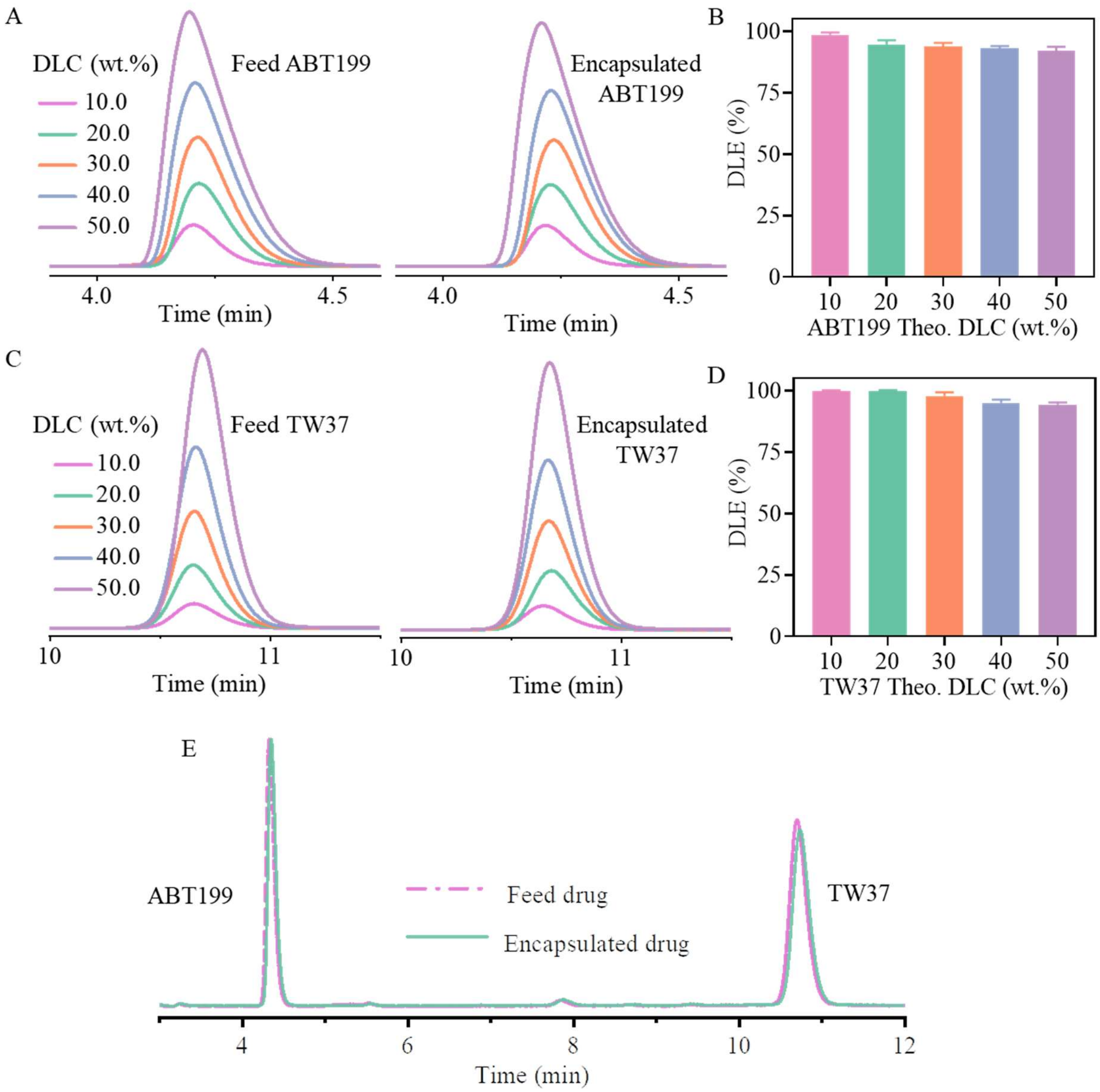

Figure S3. Representative HPLC chromatogram of ABT199 and TW37 loaded in nanoparticles at different drug loading contents. (A) Feed and encapsulated ABT199 for NPA. (B) DLE changes of ABT199 in NPA with theoretical DLC (n = 3). (C) Feed and encapsulated TW37 for NPT. (D) DLE changes of TW37 in NPT with theoretical DLC (n = 3). (E) Feed and encapsulated ABT199 and TW37 for NPAT.

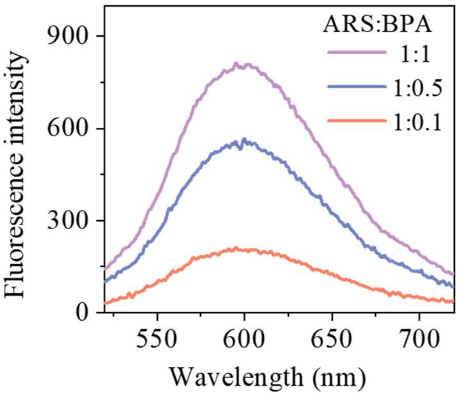

Figure S4. Fluorescence spectra of ARS/PEG-*b*-P(BPA-*co*-Tyr) complexes at different ARS/BPA molar ratios.

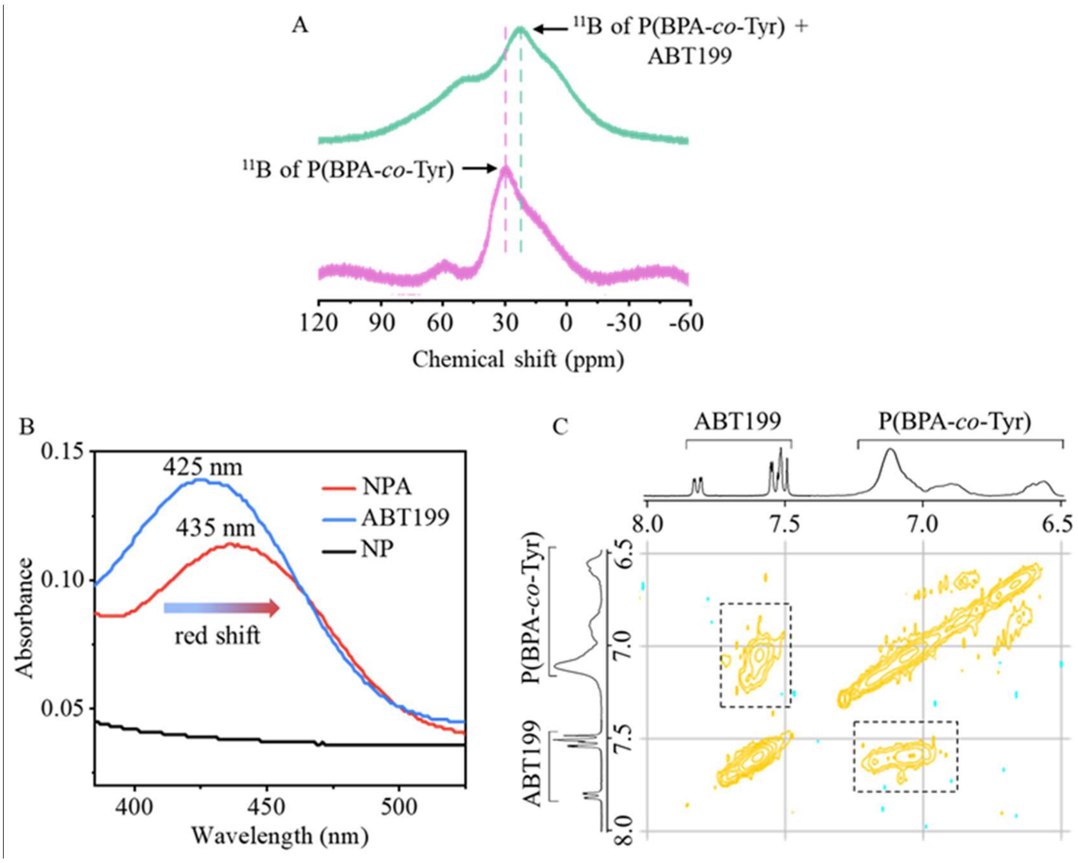

Figure S5. Investigation of the B-N coordination and π-π stacking between ABT199 and P(BPA-*co*-Tyr) segments in copolymers. (A) ^{11}B NMR spectra of PEG-*b*-P(BPA-*co*-Tyr) before and after mixing with ABT199 in DMSO-d_6. (B) UV-vis spectra of NPA, ABT199, and NP in PBS (pH 7.4). (C) 2D ^{1}H-^{1}H nuclear overhauser effect spectroscopy (NOESY) of NPA in DMSO-d_6/D_2O (v/v, 9:1, 6.5-8.0 ppm).

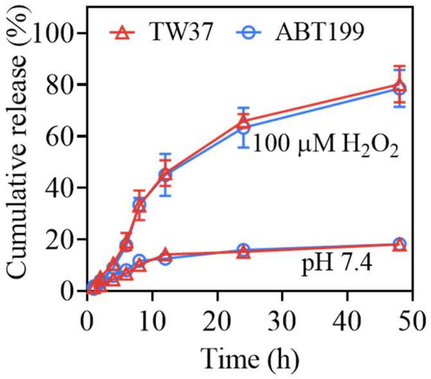

Figure S6. *In vitro* TW37 and ABT199 release profiles from NPAT in PBS (pH 7.4, 10 mM) containing 100 μM H_2O_2 (n = 3).

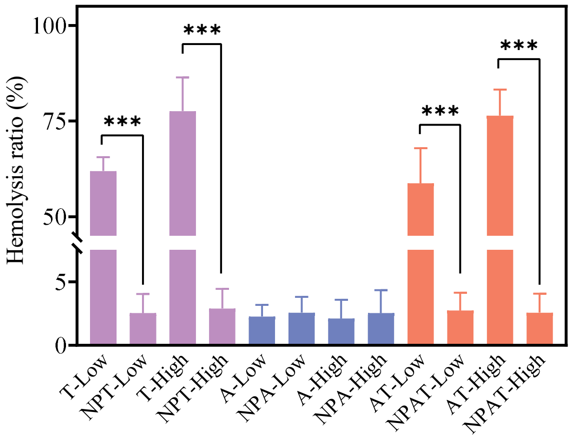

Figure S7. Hemolysis rate of erythrocytes treated with different formulations. Statistical analysis was performed by two-tailed Student's *t* test, n = 3, ***$p < 0.001$.

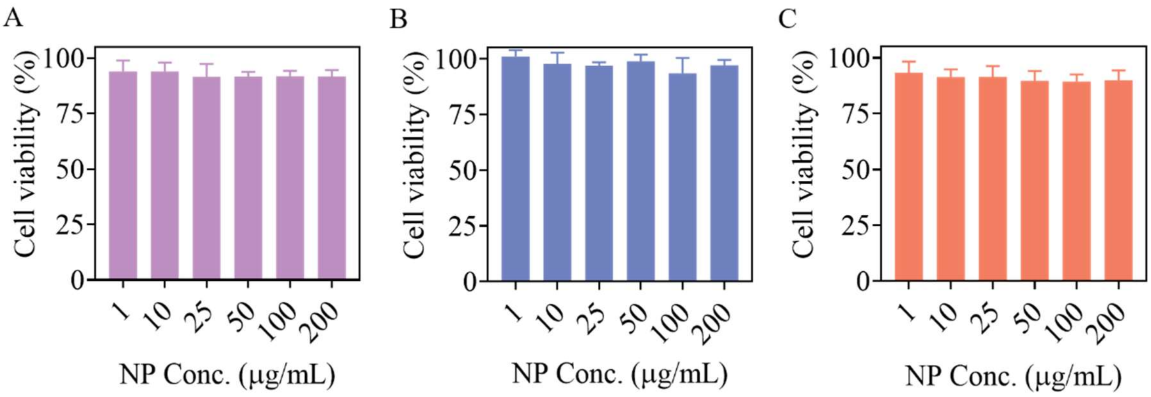

Figure S8. Cell viability of (A) L929 cells, (B) MOLM-13-Luc cells, and (C) MV-411 cells following 48 h treatment with blank nanoparticles (n = 6).

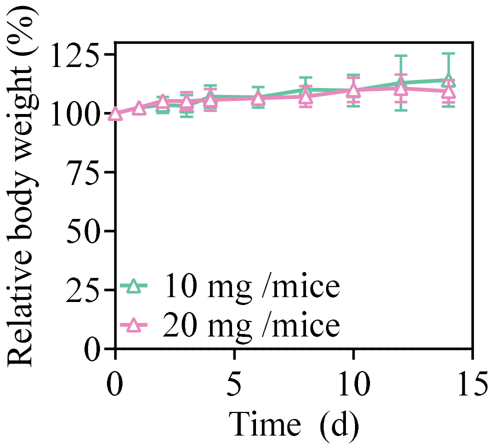

Figure S9. Body weight changes at various NP dosages (n = 4).

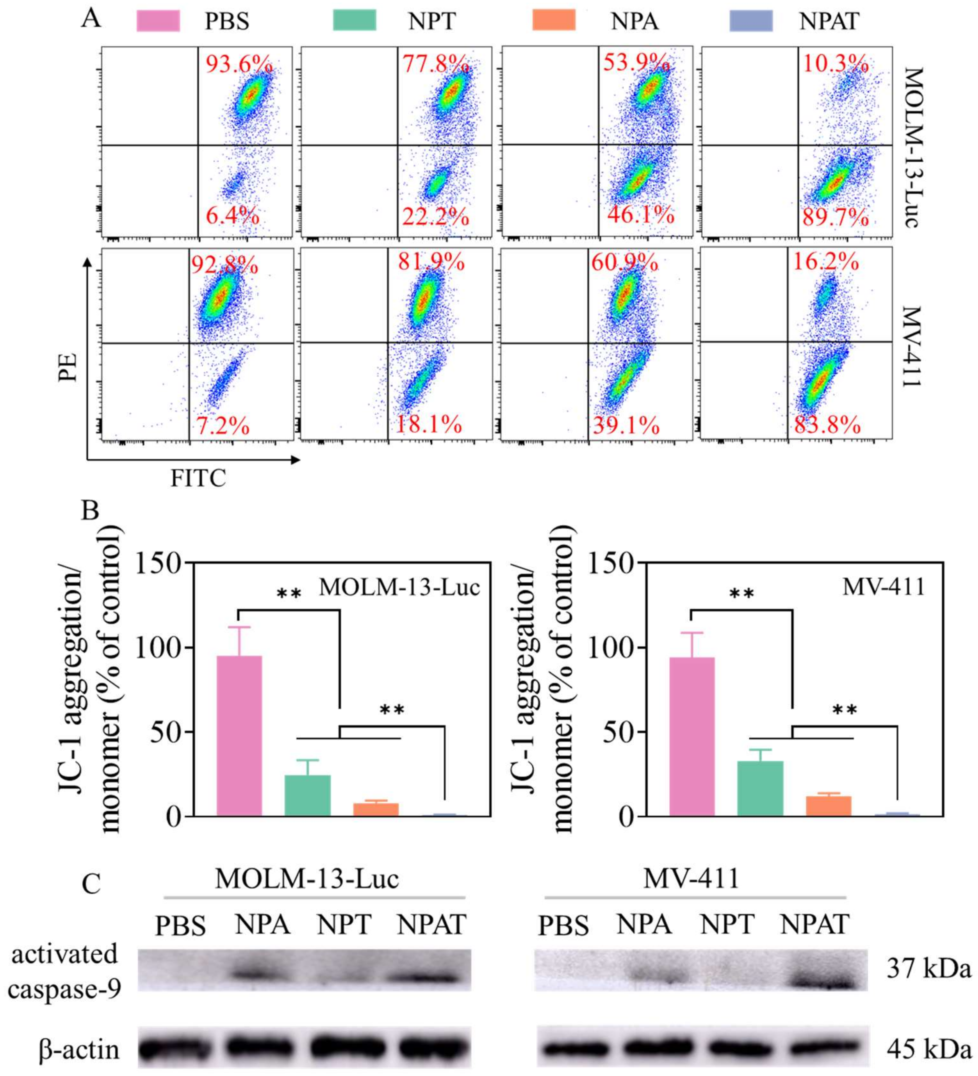

Figure S10. (A) Flow cytometric analysis of JC-1 aggregation (PE) and monomer (FITC) in both MOLM-13-Luc and MV-411 cells treated with NPT, NPA or NPAT for 48 h. (B) Quantitative analysis of JC-1 aggregation/monomer ratios. The statistical significances were assessed by two-tailed Student's *t* test, n=3, $^{**}p < 0.01$. (C) The expression of activated caspase-9 in MOLM-13-Luc and MV-411 cells treated with NPA, NPT or NPAT for 48 h. The drug concentrations used for MOLM-13-Luc and MV-411 cells were 10 ng/mL and 60 ng/mL, respectively.

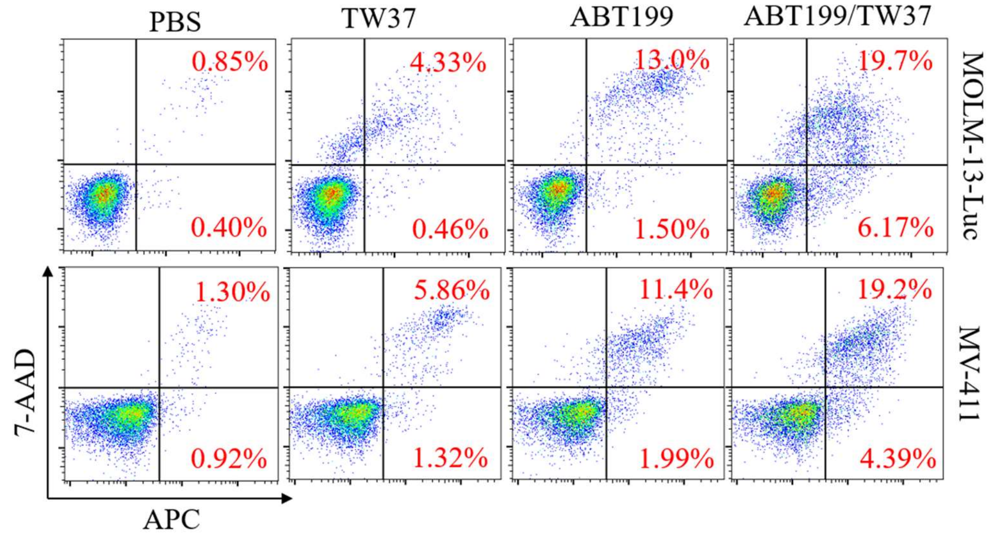

Figure S11. Apoptosis of MOLM-13-Luc (ABT199: 10 ng/mL, TW37: 10 ng/mL) and MV-411 (ABT199: 60 ng/mL, TW37: 60 ng/mL) cells induced by free drugs for 48 h.

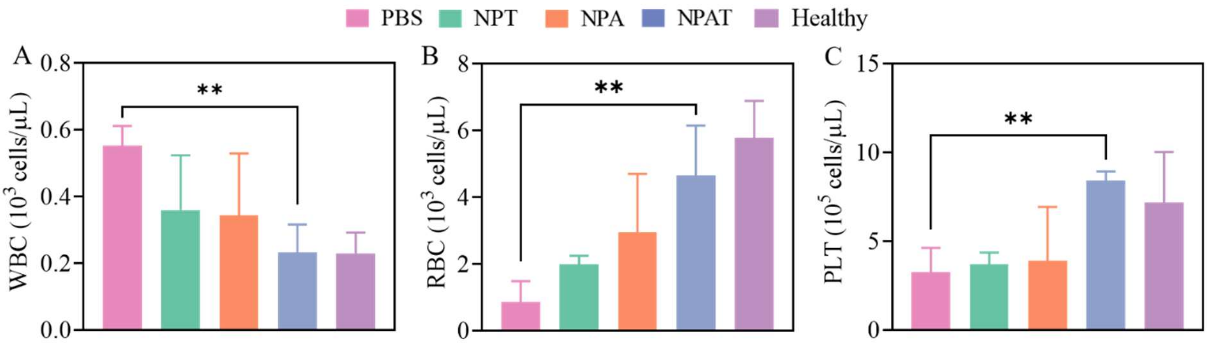

Figure S12. Blood routine test. The blood was collected from celiac arterial blood on day 14. (A) white blood cells (WBC), (B) red blood cells (RBC), and (C) platelets (PLT). Statistical analysis was performed by two-tailed Student's *t* test, n = 3, ***p* < 0.01.

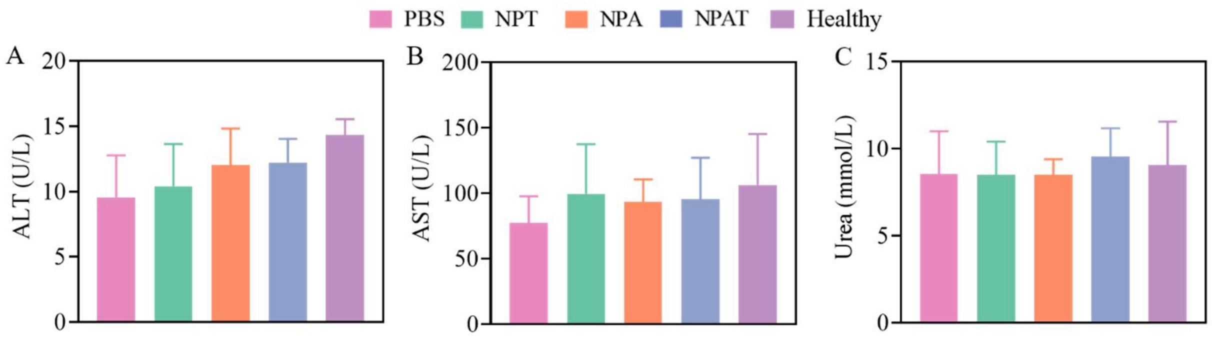

Figure S13. Blood biochemical test, n = 3. The blood was collected from celiac arterial blood on day 14. (A) alanine aminotransferase (ALT), (B) aspartate aminotransferase (AST), and (C) urea.

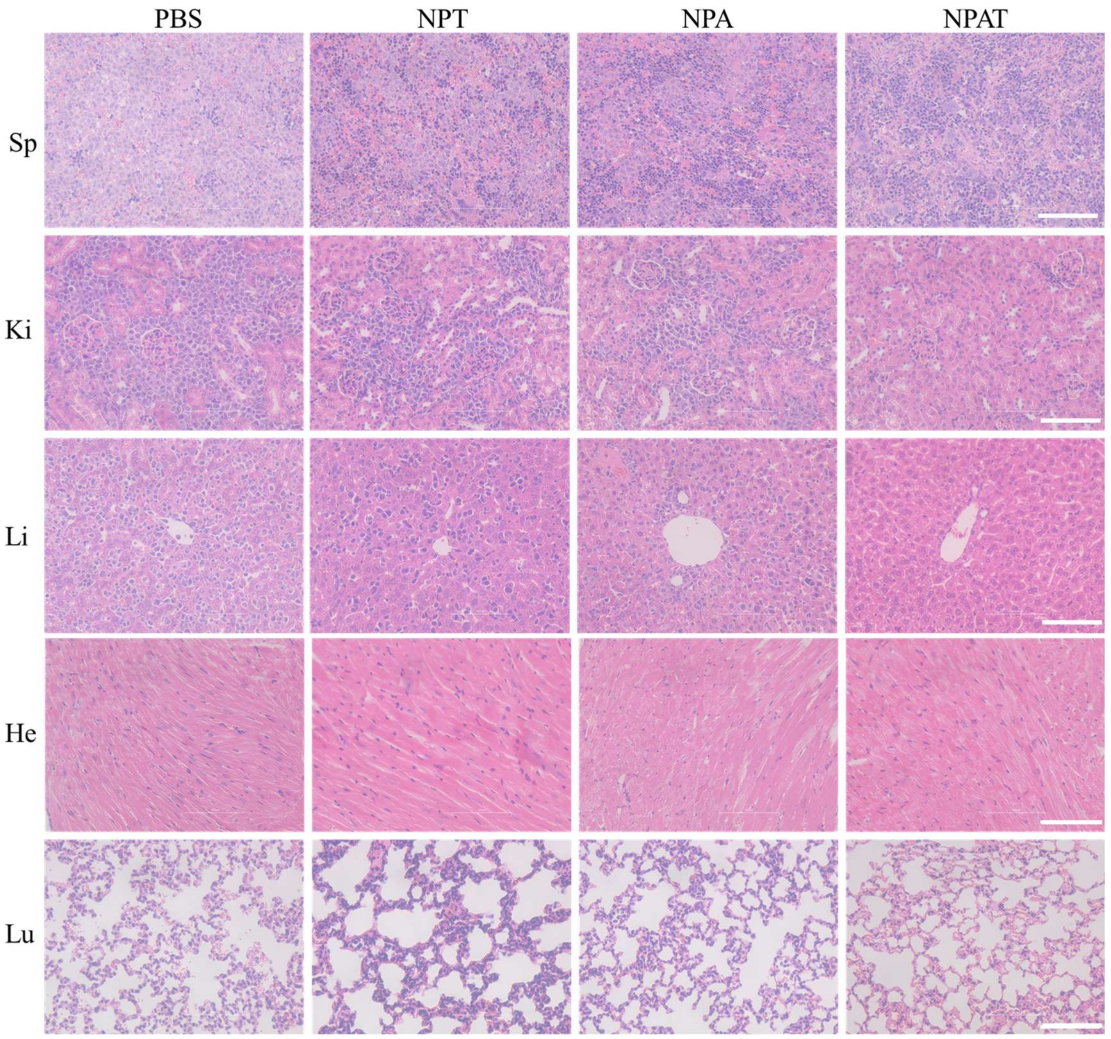

Figure S14. Cell invasion in spleen (Sp), kidey (Ki) liver (Li), heart (He) and lung (Lu) characterized by H&E staining. Scale bar: 100 µm.

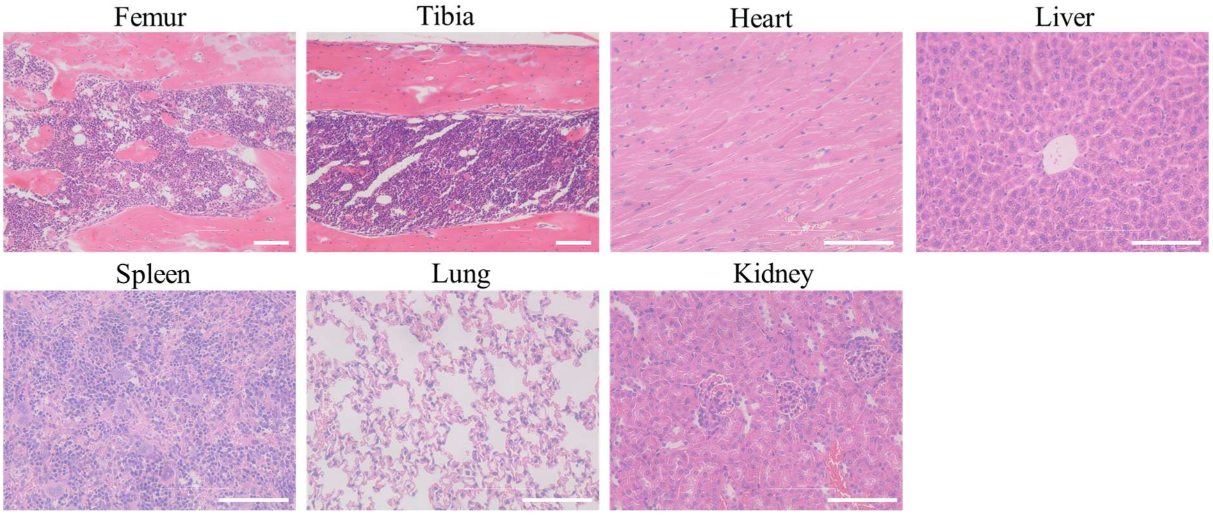

Figure S15. H&E staining of femur, tibia, and main organs from healthy mice. Scale bar: 100 μm.

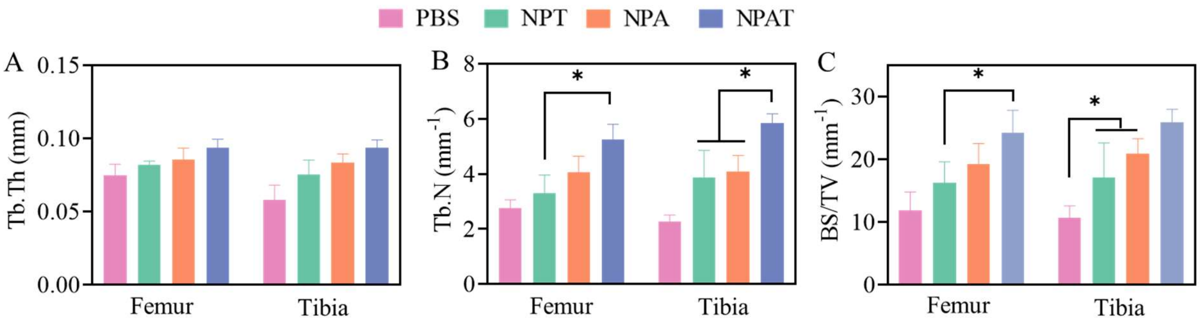

Figure S16. Quantitative analysis of trabecular thickness (Tb.Th), trabecular number (Tb.N), and bone surface/tissue volume (BS/TV). Statistical analysis was performed by two-tailed Student's *t* test, n = 3, *$p < 0.05$.

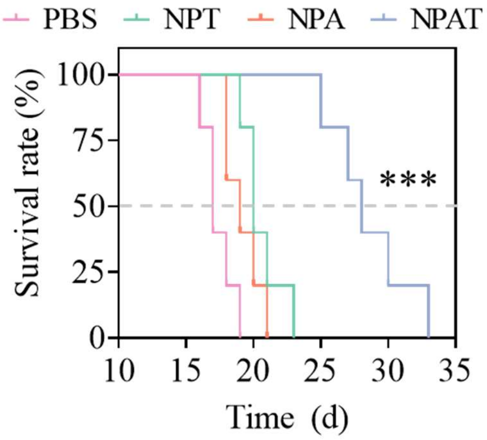

Figure S17. Survival curves of mice in different treatment groups. NPAT vs PBS, NPT, and NPA. Statistical analysis was performed by log-rank Mantel-Cox test, n = 5, ***$p < 0.001$.
